# Overlooking early glaucoma with an apparently normal OCT RNFL: beware of “Green Disease”

**DOI:** 10.1038/s41433-025-03661-0

**Published:** 2025-02-10

**Authors:** Malachy Nemet, Polina Lankry, Michael Waisbourd

**Affiliations:** 1 School of Medicine, Faculty of Medical and Health Sciences, Tel Aviv University, Tel Aviv, Israel; 2 Department of Ophthalmology, Tel-Aviv Medical Center, Tel-Aviv, Israel

**Keywords:** Glaucoma, Ocular hypertension

Glaucoma involves both structural and functional impairments, necessitating an integrated approach to evaluate disease progression. Structural atrophy of the retinal nerves and functional visual field (VF) loss are key indicators of glaucomatous damage. Early structural changes in the ganglion cell layer (GCL) and retinal nerve fiber layer (RNFL) can be detected using high-resolution optical coherence tomography (OCT) [1], while VF testing reveals early functional defects [2]. However, despite advancements in OCT technology, limitations in its interpretation remain a significant challenge.

OCT uses color-coded maps to categorize RNFL thickness values: white and green indicating normal ranges, yellow for borderline, and red for below-normal levels. While these maps provide quick visual guidance, overreliance on them can lead to missed early glaucomatous changes, a phenomenon termed “green disease” [3–5]. Variations in normative databases, device calibration, and patient demographics contribute to this issue, as subtle structural changes may not trigger abnormal color coding. Clinicians should look beyond the color-coded 4-quadrant RNFL maps to avoid underdiagnosis.

A more effective diagnostic approach involves detailed evaluation methods that incorporate OCT RNFL deviation maps, GCL analysis, and VF testing. For example, RNFL deviation maps can highlight localized defects that standard quadrant maps miss, such as wedge defects. GCL analysis offers additional structural insights, particularly for early-stage glaucoma, where changes in the macular region often precede RNFL thinning. Correlating these structural findings with VF defects further improves diagnostic accuracy.

Consider the case of a 67-year-old woman with a family history of glaucoma. Her OCT RNFL quadrant maps appeared “normal,” but the RNFL deviation map revealed a subtle wedge defect. Corresponding VF testing confirmed a very early superior nasal step, and GCL analysis supported the presence of early structural damage. This integration of findings led to a diagnosis of normal-tension glaucoma, underscoring the limitations of relying solely on color-coded maps (Fig. 1).

**Fig. 1:**
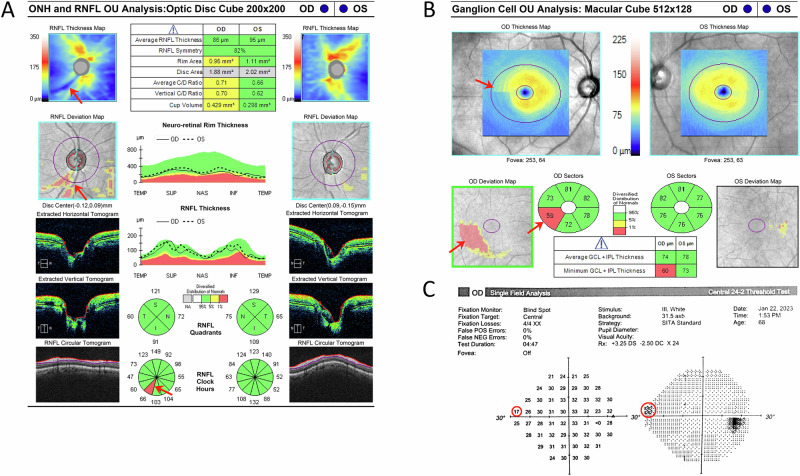
Early glaucomatous damage with apparently “normal” OCT RNFL quadrant pie chart. Retinal nerve fiber layer (RNFL) wedge defect in the right eye is evident in the optical coherence tomography (OCT) thickness map, but could be easily missed in the 4-quadrant pie chart (**A**). Corresponding defects appear in the ganglion cell layer (GCL) OCT and Humphrey visual field test (**B**, **C**).

In another instance, a 53-year-old woman with optic disc cupping and an inferior Drance hemorrhage, presented another diagnostic challenge. While her OCT RNFL thickness was within normal limits on the quadrant map, deviation maps revealed an inferior wedge defect. VF testing identified a superior nasal step, which correlated with RNFL thinning and confirmed early glaucomatous damage. This case highlights the importance of detailed deviation map analysis to detect localized defects (Fig. 2).

**Fig. 2:**
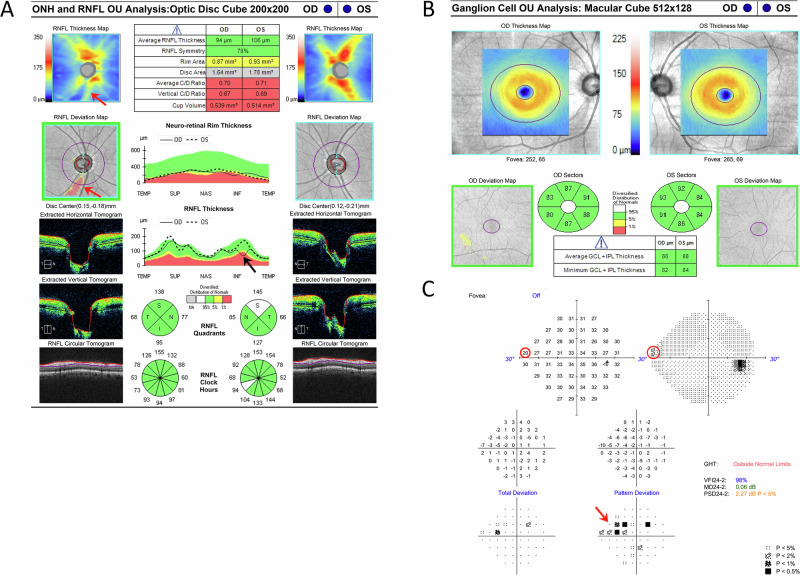
Subtle OCT changes that could be easily missed, suggesting very early glaucoma. Retinal nerve fiber layer (RNFL) wedge defect in the right eye is evident in the optical coherence tomography (OCT) thickness map (**A**), but could be easily missed in the 4-quadrant pie chart and ganglion cell layer (GCL) OCT (**B**). Corresponding defects appear in the Humphrey visual field test (**C**).

These examples underscore the limitations of relying solely on color-coded RNFL maps. While these maps are useful screening tools, they should not replace a detailed assessment of OCT deviation maps, GCL changes, and VF testing. Recognizing “green disease” and integrating these additional diagnostic tools into routine practice can significantly enhance glaucoma diagnosis and management. By adopting a holistic approach, clinicians can identify subtle glaucomatous damage earlier, improving outcomes and preventing progression.

In conclusion, while advancements in OCT technology have revolutionized glaucoma care, they also demand a nuanced understanding of its limitations. Clinicians should prioritize comprehensive evaluation strategies that move beyond color-coded maps to detect subtle, early glaucomatous changes. Recognizing and addressing “green disease” is essential for accurate diagnosis, timely intervention, and improved patient outcomes.

## Data Availability

The data supporting this study are available upon reasonable request to the corresponding author.
